# Modeling Some Possible Handling Ways with Fish Raw Material in Home-Made Sushi Meal Preparation

**DOI:** 10.3390/foods8100459

**Published:** 2019-10-08

**Authors:** Hana Buchtova, Dani Dordevic, Iwona Duda, Alena Honzlova, Piotr Kulawik

**Affiliations:** 1 Department of Meat Hygiene and Technology, Faculty of Veterinary Hygiene and Technology, University of Veterinary and Pharmaceutical Sciences Brno, 61242 Brno, Czech Republic; 2 Department of Plant Origin Foodstuffs Hygiene and Technology, Faculty of Veterinary Hygiene and Technology, University of Veterinary and Pharmaceutical Sciences Brno, 61242 Brno, Czech Republic; dordevicd@vfu.cz; 3 Department of Technology and Organization of Public Catering, South Ural State University, Lenin prospect 76, 454080 Chelyabinsk, Russia; 4 Department of Animal Product Technology, Faculty of Food Technology, University of Agriculture, 31-120 Krakow, Poland; iwona.duda@urk.edu.pl (I.D.); kulawik.piotr@gmail.com (P.K.); 5 Department of Chemistry, State Veterinary Institute Jihlava, 58601, Jihlava, Czech Republic; honzlova@svujihlava.cz

**Keywords:** nigiri sushi, polycyclic aromatic hydrocarbons, histamine, household smoker unit

## Abstract

The aim of this work was to simulate selected ways of handling with raw fish after its purchase. The experiment was designed as three partial simulations: (a) trend in the biogenic amines formation in raw fish caused by breakage of cold chain during the transport after purchase, (b) the use of a handheld gastronomic unit as an alternative method of smoking fish with cold smoke in the household with regard to a possible increase in polycyclic aromatic hydrocarbon content, and (c) whether the cold smoked fish affects selected sensory parameters of nigiri sushi meal prepared by consumers. The material used in the research consisted of: yellowfin tuna (*Thunnus albacares*) sashimi fillets and the Atlantic salmon (*Salmo salar*) fillets with skin. The control (fresh/thawed tuna; without interrupting the cold chain) and experimental (fresh/thawed tuna; cold chain was interrupted by incubation at 35 °C/6 h) samples were stored at 2 ± 2 °C for 8 days and analyzed after 1st, 4th and 8th day of the cold storage. Histamine content was very low throughout the experiment, though one exception was found (thawed tuna without interrupting the cold chain: 272.05 ± 217.83 mg·kg^−1^/8th day). Tuna samples contained more PAH (4.22 µg·kg^−1^) than salmon samples (1.74 µg·kg^−1^). Alarming increases of benzo(a)anthracene (1.84 μg·k^−1^) and chrysene (1.10 μg·kg^−1^) contents in smoked tuna were detected.

## 1. Introduction

Currently, sushi meals are becoming popular worldwide [1]. Sushi meals have developed from a simple street food to sophisticated cuisine. Many studies have dealt with the health benefits and health hazards associated with the sushi cuisine [2]. In the past, high attention has been devoted to studies of microbiological [3], chemical [4,5] or parasitic [6] hazards in fishery products, like the toxicological risks of diseases after consumption of raw fish or foodstuffs that include raw fish flesh [1]. Recorded cases of acute gastric anisakiasis are a serious warning to consumers [7]. Sushi belongs to ready to eat foods and is predisposed to contamination with food pathogens, such as *Listeria monocytogenes* [8,9].

Consumer concerns about food safety might disrupt a healthy food choice [10]. Risks associated with the consumption of fish might impose barriers to consumption, though fish is considered an important component of the human diet [11].

A new look at research on sushi meal assessment, including simulation of model of real consumer behavior and culinary practices by chefs, should shift research to a higher level of knowledge. In recent years there has been an increase in collaboration between researchers and chefs in the field of gastronomy [12]. Modern trends of molecular gastronomy that works with human senses, is a fast food preparation method using portable, easy-to-use applications, developed specifically for chefs, to create new unusual flavors.

The biggest interruption in the cold chain occurs after product purchase and during its delivery to the household. Consumer behavior and the ambient temperature largely influence the shelf life and food safety [13].

In recent years, also in the Czech Republic, there has been a growing trend of self-preparation of sushi food by consumers. In our research, based on the buying habits of some consumers and their creativity approach to treat raw fish raw using a handheld smoker unit, we wanted to connect partial studies on fish handling and sushi preparation into one model experiment.

The experiment was designed in the form of three partial simulations: (a) trend in biogenic amines formation caused by severe breakage of cold chain during the transport of fish raw material after purchase to household, (b) the use of a handheld gastronomic unit as an alternative method of smoking fish with cold smoke in the household with regard to a possible increase in polycyclic aromatic hydrocarbons (PAH) content, and (c) whether the cold smoked fish affects selected sensory parameters of nigiri sushi meal prepared by consumers in their households.

## 2. Materials and Methods

Fresh sashimi fillets of the yellowfin tuna (*Thunnus albacares*, caught, FAO 71 area, category of fishing gear: seines) and fresh fillets with skin of the Atlantic salmon (*Salmo salar*, farmed, Norway) were bought from a retail shop (Ocean48, Brno, Czech Republic).

### 2.1. Trend in the Biogenic Amines Formation Caused by Severe Breakage of Cold Chain during the Transport of Fish Raw Material after Purchase Place to Household

Tuna sashimi fillet was used to a case study focused on simulating conditions sale of fresh and thawed fish and consumer behavior (compliance/interruption with/of the cold chain) and to determine how this behavior affects the formation of biogenic amines total content and its spectrum (tryptamine TRP, 2-phenylethylamine 2-PHE, putrescine PUT, cadaverine CAD, histamine HIS, tyramine TYR, spermidine SPD, spermine SPR). The experiment was carried out in four separate replicates. Tuna fillets purchased for the fifth repetition had to be excluded from the experiment because of the high histamine content at the start of the storage, which significantly exceeded the limit set out in Regulation (EC) No 2073/2005 [see in Appendix A1] at the start of storage (the possible reason for higher histamine content will be commented on in the Results and Discussion section).

The case study was based on the use of four different sashimi fillets (1, 2, 3, 4). Four types of samples A, B, C, D from each tuna fillet were prepared simultaneously. Characteristics of the samples were as follows: A: control sample, fresh tuna was cold stored at +2 ± 2 °C, without interrupting the cold chain after buying the fish in a store; B: experimental sample, fresh tuna, cold chain of fresh sample was interrupted before the cold storage in a laboratory by incubation of the sample (35 °C/6 h) to simulate the possible consumer behavior in the summer after buying the fish in a store, subsequently samples were cold stored at +2 ± 2 °C; C: experimental sample, thawed tuna, after buying the fish in a store the sample was experimentally frozen (−35 °C) and stored for two weeks in a frozen state (−18 °C), then the samples were thawed in the refrigerator (+2 ± 2 °C/12 h) and subsequently cold stored at +2 ± 2 °C; D: experimental sample, thawed tuna, after buying the fish in a store, the sample was experimentally frozen (−35 °C) and stored two weeks in a frozen state (−18 °C), the cold chain of thawed sample was interrupted by incubation the sample (35 °C/6 h) to simulate the possible consumer behavior in the summer after buying the fish in a store, the samples were subsequently cold stored at +2 ± 2 °C. All samples (A, B, C, D) were stored at +2 ± 2 °C for 8 days, the samples were analyzed after 1st, 4th and 8th days of cold storage.

The biogenic amines analysis was performed according to the method described by [14]. The chromatographic separation was performed using a Dionex Ultimate 3000 HPLC apparatus (Thermo Scientific, Waltham, MA, USA) with a FLD 3400RS four channel fluorescent detector (Thermo Scientific) and a low pressure gradient pump with a four channel mixer. The detector settings were set to 340 nm for excitation and 540 nm for emission. The separation was performed on a Kromasil 100-5-C18 4.6 × 250 mm column (Akzo Nobel, Amsterdam, The Netherlands) and the column temperature of 30 °C. Flow rate was 0.8 mL/min with two mobile phases: (A) acetonitrile (Merck, Darmstadt, Germany) and (B) water (Merck). The detection limit for each biogenic amine was 0.005 mg·kg^−1^. The samples were analyzed in duplicate and triplicate and injections into HPLC were carried out on each duplicate (N = 4 × 2 × 3).

### 2.2. The Part of the Research Consisting of Testing the Use of a Handheld Gastronomic Unit as an Alternative Method of Smoking Fish With Cold Smoke in the Household with Regard to a Possible Increase in Polycyclic Aromatic Hydrocarbons (PAH) Content and Whether the Cold Smoked Fish Affects Selected Sensory Parameters of Nigiri Sushi Meal Prepared by Consumers at Their Household

Tuna and salmon fillets were used for preparation of nigiri sushi with not-smoked (raw, control samples) and smoked (experimental samples) samples. Smoked muscle of both fish was prepared with application of a smoker unit (Super Aladin smoker, Manihi s.r.o., Praha, Czech Republic). Cold smoke (20 °C; Aladin oak chips) was applied on meat surface beneath the glass hatch for 5 min. The experiment was carried out in five separate replicates. Sensory attributes (saltiness, bitterness, juiciness, consistency) of sushi meal and a question focused on examining the fact whether the conscious consumption of smoked fish in sushi can affect the consumer’s confidence in the health safety of this food were monitored by a group of trained evaluators and evaluated on the basis of questionnaires.

**Figure 1:**
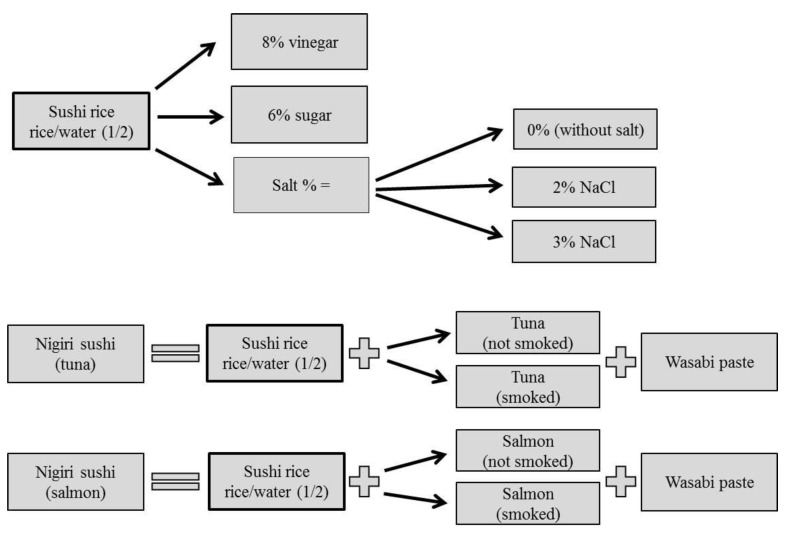
Experimental design of sushi production samples used in the research.

Nigiri sushi samples with tuna and salmon meat (smoked and not smoked) were prepared in the Sensory Laboratory at the Department of Meat Hygiene and Technology (Faculty of Veterinary Hygiene and Ecology, University of Veterinary and Pharmaceutical Sciences, Brno, Czech Republic). Fillets were frozen according to the Commission regulation (EC) No 853/2004 (Annex III, Section VIII, Chapter III, Part D, Point 2a [see in Appendix A2]) at −40 °C using quick freezing unit F.R.C. BF 031AF (Friulinox, Taiedo di Chions, Italy) to muscle core temperature of −20 °C and were stored in a frozen chamber with regulated temperature (−20 ± 2 °C) for 2 weeks. Then the samples were thawed in refrigerator (+2 ± 2 °C/12 h) and subsequently divided into two parts, the one was used as control (raw not smoked) samples for sushi preparation and the second one was used for cold smoking by Super Aladin smoker and for sushi preparation. The rice was cooked in rice cooker (42507 Design Reiskocher, Gastroback, Hollenstedt, Germany). Information about sushi ingredients are following: sushi rice (short grain variety, Yutaka, Italy), 8% vinegar (apple vinegar, Bzenecky Ocet, Bzenec, Czech Republic), 6% sugar (sugar crystal, producer: Korunni, Hrušovany nad Jevišovkou, Czech Republic) and 2% cooking salt with iodine (NaCl 98%, J 20-34 mg·kg^−1^, K+S Czech Republic a.s., Olomouc, Czech Republic), wasabi paste (Yutaka, China). The experimental design is shown in Figure 1.

Sushi samples were sensory evaluated in the laboratory equipped according to ISO 8589:2008 [see in Appendix A3]. The protocol consisted of unstructured graphical scales of 100 mm length, with one edge of the scale representing the strongest expressed attribute and the second one the weakest expressed attribute. Saltiness (2% salt addition) was evaluated by respondents’ comparison with sushi samples prepared without salt (0 point) and with 3% salt content (100 points) added to rice.

Twenty panelists took part in the sensory evaluation, where they assessed selected parameters: (saltiness, bitterness, juiciness, consistency) and consumer confidence in the food safety (expressed in their own words). Ingredients’ weights (g) of nigiri sushi are given in Table 1.

The thawed raw tuna and salmon samples were used for chemical analysis (total protein, total fat and dry matter content). The same thawed not smoked and smoked tuna and salmon samples were used for determination of polycyclic aromatic hydrocarbons (PAH).

The total protein content (ISO 937:1978 [see in Appendix A4]) was determined as the amount of organically bound nitrogen (recalculating coefficient *f* = 6.25) using the analyzer Kjeltec 2300 (FOSS Tecator, Höganäs, Sweden). The total lipid content was determined quantitatively (ISO 1443:1973 [see in Appendix A5]) by extraction in solvents using Soxtec 2055 (FOSS Tecator). The dry matter was determined gravimetrically according to the Czech National Standard (ISO 1442:1997 [see in Appendix A6]) by drying the sample to a constant weight at +103 ± 2 °C (Binder FD 53, Tuttlingen, Germany).

PAH were determined by accredited method (no. 19 Standard operating procedure 8.15A) using HPLC/FLD in the laboratory of State Veterinary Institute Jihlava (Jihlava, Czech Republic). Each sample was analyzed in parallel. A thoroughly homogenized samples, after trituration with anhydrous sodium sulphate p.a. (Lach-Ner, sro, Tovarni 157, 27711, Neratovice, Czech Republic) and after addition of internal standard 2-methylchrysene (Dr. Ehrenstorfer GmbH, Augsburg, Germany) were extracted with diethyl ether. Extracts were filtered through glass fiber filter paper (Cat. No. 516-0867, VWR International bvba, Leuven, Belgien, the solvent was evaporated on a Büchi R-134 rotary evaporator (BÜCHI Labortechnik) AG, Flawil, Switzerland) at a maximum temperature of 30 °C. The residue was carefully blown off with a stream of nitrogen. The extracted fat was dissolved in chloroform (Cat. No. 20034-UT2-M2500-7 (Macron 6754), Lach-Ner, Ltd., Neratovice, Czech Republic). An aliquot of the solution was purified by gel permeation chromatography on a Gilson Aspec XL system (Gilson, Middleton, WI, USA) using a PAH prep column (500 × 8 mm) packed with gel (styrene divinylbenzene copolymer) (Watrex Praha, sro, Carolina Center, Prague, Czech Republic). Purified samples were evaporated to near dryness on a Büchi R-134 rotary evaporator at a maximum temperature of 30 °C or the remaining solvent was blown off with a stream of nitrogen. The residue was dissolved in acetonitrile and used for fluorescence detection by liquid chromatography.

Chromatographic analysis was performed on Waters Alliance e2695 liquid chromatograph with 2475 fluorescence detector (Waters Corporation, Milford, MA, USA) on Waters PAH column (250 mm × 4.6 mm × 5 µm) using gradient elution with mobile phase. Gradient mobile phase consisted out of acetonitrile/redistilled water (75/25 100/0), flow rate 0.7 mL/min, injection 10 μL, column temperature 30 °C. Detection was performed by fluorescence detector with programmable wavelength change (excitation wavelength—265 nm for benzo(a)anthracene and chrysene, 290 nm for benzo(b)fluoranthene and benzo(a)pyrene; wavelength 380 nm for benzo(a)anthracene and chrysene and 430 nm for benzo(b)fluoaranthene and benzo(a)pyrene). PAH Calibration Mix, CRM47940 (Supelco Analytical, Bellefonte, PA, USA) was used for the calibration. The limit of quantification was 0.25–0.29 µg/kg, the repeatability of the method was 10% and yield 65–95%.

### 2.3. Statistical Analysis

The results of chemical composition were evaluated (mean ± s. d.) in the program Microsoft Office Excel 2007 (Microsoft Corp., Redmond, WA, USA). Statistically significant differences of biogenic amines (BA) spectrum were performed at levels of α = 0.05 (*p* ˂ 0.05) using the UNISTAT 6.0 (Unistat^®^ Ltd, London, UK) statistical package (multiple comparison, Tukey’s HSD test).

## 3. Results and Discussion

Fresh fishery products are among the most perishable food commodities Their quality is influenced by a number of factors (the origin-wild/farmed, water temperature and level of environmental pollution, compliance with veterinary and hygienic standards during hunting and after capture, species and health and nutritional status/age/sex/phase of sexual cycle/size/weight of fish, way of treatment-gutting/cutting, initial microbiological contamination, keeping the cold chain) which can vary in time and therefore consequently the quality can vary significantly from batch to batch [15]. Freezing is an excellent way to extend shelf life of fish meat during long-term transport or frozen storage [16]. Offering thawed fishery products is a common way of selling fish in landlocked countries [17]. On the other hand, cold chain breakage can cause potentially serious alimentary intoxication, such as histamine content increment. Therefore, growth and activity of histamine-producing bacteria can become dangerous especially in fish which muscle tissue contain a high concentration (about 10,000 mg·kg^−1^) of free histidine in muscle tissue as tuna fillets. Though, salmon has a low concentration (about 100 to 200 mg·kg^−1^) of free histidine [18]. Especially, *Photobacterium phosphoreum* and psychrotolerant bacteria similar to *Morganella morganii* are known to be present in fresh fish tissue and form histamine at low temperatures (under 5 °C) [19]. Appearance freshness is often unlawfully restored by an injection or immersion of fillets in the nitrites solution to change the dark red or brown colour (visually less fresh meat) to red pigmentation. Meat after this application looks fresh but histamine levels can be high. Also, inappropriate practices of freezing with subsequent illegal treatment are often used (tuna fish originally of canning grade can be illegally sold as sushi grade of tuna). According to the regulation (EC) no 853/2004 [see in Appendix A2], fishery products shall be frozen below −18 °C, for canning industry; unprocessed fish initially is tolerated to be frozen in brine at −9 °C. Subsequent illegal treatment of tuna fillets and application of nitrites/nitrates (e.g., salt, additives or vegetable extracts containing high level of nitrites) or using of gas carbon monooxide (CO) is not authorized according to the regulation (EC) no 1333/2008 [see in Appendix A7]. Approximately 25,000 tons of tuna per year undergo this treatment [20].

Besides the fact that consumers may be deceived by the quality of fillets, their health can also be compromised. The high level of histamine can cause allergic syndrome [21], nitrites may lead to formation of nitrosamines that have carcinogenic effects [22]. As we wrote in the Materials and Methods section, fresh tuna fillets purchased for the fifth replicate of the experiment, could be hypothetically treated by any of the above illegal practices. Fresh/thawed samples were stored at +2 ± 2 °C; the samples contained in the 1st day of 781/195, 4th day of 1851/1542 and 8th day of 1717/2914 mg·kg^−1^ of histamine in meat. This fifth fillet was therefore excluded from the further experimentation. However, we have shown that even frozen tuna fillet can become a serious threat to human health due to the increment of BA.

### 3.1. Trends in Biogenic Amines Formation Caused by Severe Breakage of the Cold Chain during the Transport to Households of Fish Raw Material after Purchase

Significant qualitative and quantitative variability in the observed BA was found between sample groups (A, B, C, D) depending on the sampling day (Table 2) as well as between the individual sampling days within one particular group (Table 3).

Regarding the criteria for foodstuffs (Regulation (EC) No 2073/2005, Chap. I, Point 1.26 [see in Appendix A1]), histamine content was very low throughout the experiment, with one exception (C/8 day) which will be further commented (Table 2). The fresh sample of tuna (A) contained the highest (*p* < 0.05) histamine content in the samples measured after 4 days (Table 3).

The fresh samples (group B) that were exposed to 35 °C/6 h and subsequently cold-stored at +2 ± 2 °C during 8 days had almost the same histamine contents during the storage period (Table 3). Distinctly higher histamine contents were found in C samples (thawed/subsequently cold-stored at +2 ± 2 °C), after 8th day of storage (the mean of four different batches: 272.05 ± 217.83 mg·kg^−1^). The noticeably high value of the standard deviation (s. d.) draws attention to possible differences in quality among purchased lots of fillets; due to these differences the results of thawed fillets (C) obtained for the 8th day of sampling are presented in Table 4. Based on the partial results for each of the four lots, it can be concluded that the three lots were probably more contaminated with microorganisms capable to decarboxylation activity. Due to their activities, histamine content of three tuna lots (8 days of storage) increased to 354.74 ± 185.67 mg·kg^−1^; PUT, CAD and TYR contents were significantly (*p* < 0.05) higher and caused five times higher total biogenic amine content (563.32 ± 252.61 mg·kg^−1^) for these three tuna lots compared to the fourth one (Table 4).

In the fourth lot, the HIS content (23.98 ± 0.93 mg·kg^−1^) was very low, but compared to the other three lots, the fillet contained significantly more SPD, though the BA total was low overall (108.67 ± 1.67 mg·kg^−1^) (Table 4). In the thawed samples of group D tuna, that were experimentally exposed to temperatures of 35 °C/6 h and subsequently cold-stored at +2 ± 2 °C during 8 days, the HIS content remained virtually unchanged between 1 and 4 days, and we found only a significant reduction in the HIS content on day 8 (Table 3). Based on higher standard deviation (s. d.) for PUT, CAD, TYR, SPD (Table 2), differences in quality were observed for group D tuna batches (similar to the batches of group C tuna). Three batches were found to have significantly (*p* ˂ 0.05) higher amounts of CAD. Consequently, the total BA content in three batches of fillets was approximately two times higher (*p* < 0.05) in comparison to the fourth fillet (Table 4).

The total BA contents of all sample groups were less than 100 mg·kg^−1^, except for samples from group C and D/8 days (Table 2, BA sum). Each of studied sample groups (A, B, C, D) showed a different trend depending on the storage time (Table 3). BA contents in fresh (A) and thawed samples (C) without cold chain break taken on the day 1 were very low (*p* > 0.05) (Table 2).

The BA content in fresh samples (A) from day 4 was significantly higher compared to day 1, followed by a significant (*p* < 0.05) decrease in BA content on the day 8 (Table 3). Thawed samples (C) contained very low levels of BA on day 1 and day 4, while BA samples were significantly (*p* < 0.05) 13 times higher on day 8. In the group of fresh (B) and thawed samples (D), where the cold chain was experimentally broken, the BA formation dynamics was more uniform, but the trend was the opposite. Overall, the BA content of Group B samples gradually decreased (significant differences were observed between days 1 and 4, 8); in contrast, in samples of group D, BA content increased gradually (significant differences were found between days 1 and 8) (Table 3).

The spectrum of BA was formed in larger quantities (above 10 mg·kg^−1^) mainly TYR and SPD and in isolated cases PUT (C and D/8th day), CAD (D/4th and 8th day). HIS contents were already discussed (A and D/4th day, C/8th day), same as SPR (B and D/1st day, D/4th and 8th day). TRP and 2-PHE levels were very low and oscillated between 0 and 3.0 mg·kg^−1^ in individual samples. Statistically significant (*p* < 0.05) differences between the biogenic amine values are given for each group and each day of the storage in Table 3 (capital letters "*A*" to "*D*" in rows).

### 3.2. Testing of Use of a Handheld Gastronomic Unit as an Alternative Method of Smoking Fish with Cold Smoke in the Household with Regard to a Possible Increase in Polycyclic Aromatic Hydrocarbons (PAH) Content and Whether the Cold Smoked Fish Affects Selected Sensory Parameters of Nigiri Sushi Meal Prepared by Consumers at Their Household

The factors affecting food or meal acceptance among consumers have changed rapidly. Sushi is admired by many consumers worldwide due to its appearance and taste [23,24,25].

A new non-traditional or unusual treatment of meal can increase consumer interest in its taste and, maybe, in its safety. We have found no previous reports of sushi meal containing tuna or salmon smoked with cold smoke for a very short period. It is possible to predict that Super Aladin smoker will be used more frequently in the practice of molecular gastronomy or home-made sushi preparation to imitate its sensory qualities and bring it closer to that of Philadelphia rolls, which is sometimes prepared with salmon smoked with cold or hot smoke.

Appearance, touch, odor, texture and taste represent the sensory properties of foods/meals. Sensory properties are one of the main factors influencing consumers’ acceptance and purchase of meals. Health consciousness (including lowering sodium content, the presence of biogenic amines or smoked products) is another factor that can have significant influence on meal acceptance. Certain sushi ingredients (such as vinegar, wasabi and sugar) provide specific sensory properties to this meal.

The sensory properties of prepared sushi samples, smoked and not smoked, are shown in Table 5. Higher values estimated by panelists for juiciness and consistency indicate worse evaluation of these sensory properties. Bigger values for bitterness are emphasizing savoury intensity. Juiciness of sushi prepared without salt was evaluated with higher values then the rest of sushi samples, though statistical significance was not observed (*p* > 0.05).

Health aspects of certain meal are getting priority over shelf life and nutritional profile [26]. Dealing with issues concerning salt consumption is also important due to the fact that salty foods belong to the group of foods toward which consumers can develop addictive tendencies [27].

Sodium intake according to World Health Organization (WHO) should not exceed 2 g per day or 5 g of natural salt (NaCl) [28]. Worldwide salt intake exceeds this limit and ranges from 9 g/day to 12 g/day, which is equivalent to 3.6 g/day to 4.8 g/day [29].

Published information about cold-smoked tuna is sparse, though cold-smoked tuna processing is similar to that of cold-smoked salmon, in which both processing and product characteristics have been extensively studied [4,18].

Warm or hot smoked seafood is accepted and consumed by consumers due to its unique taste, texture and color. Additionally, due to dehydrating, bactericidal and antioxidant properties, smoking processes increase food shelf life. The problem with smoking of foods is that during the process of smoking considerable amount of polycyclic aromatic hydrocarbons (PAH) can be formed due to incomplete wood combustion. Phenols present in wood smoke belong to desirable molecules, since they positively affect food sensory properties and shelf life, but PAH compounds are undesirable molecules [30].

Table 6 shows contents of polycyclic aromatic hydrocarbons (PAH) in the samples of smoked and not smoked tuna and salmon. Tuna samples contained more PAH (4.22 µg·kg^−1^) than salmon samples (1.74 µg·kg^−1^).

Despite alarming content findings of B(a)A (1.84 μg·kg^−1^) and CHr (1.10 μg·kg^−1^) in smoked tuna, the content of B(a)P and sum of PAH in our samples were lower than the maximum levels written in the regulation (EC) no. 1881/2006, Annex, Section 6 (B(a)P [see in Appendix A8]: 2 µg·kg^−1^, sum of PAH: 12 µg·kg^−1^).

The existence of several factors affecting PAH content in smoked fish has been scientifically confirmed. PAHs are produced during combustion processes and smoke formation. The type of used matrix (wood), combustion temperature, smoke generation technique, filtration, temperature and smoke composition. Following factors are also influencing PAH formation: size, treatment and chemical composition of smoked fish. Regarding the effect on food (fish), the diffusion intensity of PAH below its surface into the muscle is relatively low. This fact that PAHs are mainly concentrated in the surface layers means that their content in the food is determined by food surface, same as total weight of the food. Surface/weight ratio is also probably responsible for higher levels of PAH in smoked tuna. Certainly, shape of smoked food (thickness and weight) are influencing PAH levels too. The content of PAHs is also associated with the fat content of food. Reference [31] also found that B(a)A content can differ significantly in dependence of seafood species.

Higher fat content in salmon samples influences higher PAH contents than in tuna samples [4,32,33]. The findings of these authors are not in agreement with the results of our chemical composition analysis (Table 7). However, the fat content, which we determined in our samples, did correspond to published values [34]. Fluctuations in filtering capacity of the smoker could also influence PAH amounts, though the processes of sample smoking took place in the laboratory under the same conditions (time of smoke/cover of samples with glass lid).

Beside the antibacterial and antioxidant properties of smoking that are connected with phenolic compounds present in wood smoke, from our results a negative impact of seafood smoking represented as an increase of PAH compounds can be also seen. The importance of PAH level control in food is important, since these compounds are carcinogenic, mutagenic and endocrine disrupting. PAH compounds (there are more than 660 identified PAH compounds) are produced in wood smoke during pyrolysis (depolymerisation) of lignin and then condensation of the lignin components in lignocelluloses at temperature above 350 °C [35,36]. Aside from PAH increment, smoking changes color of food due to Maillard reaction (change coloration occurs due to the reaction of carbonyl groups in smoke with amino groups present on the surface of smoked food [36]. Phenols from wood smoke enter seafood by diffusion and capillary action, changing its flavor, color and prolonging shelf life [30]. PAH compounds in canned smoked tuna were 17.67 µg·kg^−1^ [37].

## 4. Conclusions

The main finding of the research is highlighting a food safety issue that was found by the experiments with a gastronomic smoking unit due to increased amounts of polycyclic aromatic hydrocarbons. The Super Aladin smoker unit is a patented product, but the manufacturer does not comment on the PAH hazards associated with food safety in the user documentation. The producers of this smoker units should at least include in the manuals the maximum smoking time depending on the type of food and its fatness. In this way they would alert users to the possible danger, which is the adhesion of harmful PAH on smoked food. The minimum or maximum exposure time of food to smoke is not specified or restricted for specific food types. For prolonged smoking (up to 24 h!), the manufacturer recommends intermittent repeated batches of smoke under the hatch at multiple time intervals as required (optical smoke density control under the hatch). In addition to commercial hardwood chips (oak, beech, Jack Daniels), users can use other alternative matrices including aromatic oils for the development of smoke. Due to the lipophilic nature of PAH, these substances could hypothetically be added to the smoke and subsequently increase PAH level to even more harmful concentrations. Certainly, that further experiments with household gastronomic smokers, such as, Super Aladin smoker, will probably give broader and more precise picture about all possibilities and issues concerning these types of devices.

In the case of the experiment aimed at monitoring the content of biogenic amines in tuna samples, we found much more favorable results than expected. Serious interruption of thawed tuna samples cold chain after purchase did not result in an increase of biogenic amines to levels that could represent a health risk for consumers. Manufacturers inform consumers that they should store purchased fish at 0–2 °C and consume it within 2 days. According to the results obtained in our experiment, tuna samples could be considered suitable for consumption after 4 days in terms of BA content and even up to 8 days (except for thawed samples without cold chain interruption) after purchase. However, we cannot recommend this practice due to the possibility that purchased tuna could have higher histamine content developed before purchase. The risk of intoxication with histamine becomes more realistic with each new day of storage. Laboratory examinations of fish species associated with high histamine content in muscle should therefore be a normal part of quality controls by sellers so that they do not have to passively rely on written statements from fish suppliers regarding histamine content in commercial or veterinary evidences of their origin.

## Figures and Tables

**Table 1:** Ingredients’ portions (%) of nigiri sushi prepared with salmon and tuna.

Fish	Treatment	Rice	Seafood	Wasabi
Tuna	Not smoked	66.84 ± 0.53	37.24 ± 0.94	0.80 ± 0.06
	Smoked	63.02 ± 2.01	36.95 ± 1.04	0.75 ± 0.08
Salmon	Not smoked	66.76 ± 0.36	32.38 ± 0.34	0.86 ± 0.09
	Smoked	65.54 ± 0.25	33.31 ± 0.39	0.84 ± 0.07

**Table 2:** Qualitative and quantitative biogenic amine spectrum (in mg·kg^−1^) for groups (A, B, C, D) depending on the sampling day.

Day	Sample	Tryptamine	2-Phenyl-Ethylamine	Putrescine	Cadaverine	Histamine	Tyramine	Spermidine	Spermine	Sum of Bas
1st	A	0.03 ± 0.05 ^a^	1.05 ± 0.68 ^a^	3.21 ± 1.77 ^ab^	2.99 ± 1.35 ^ab^	2.00 ± 1.40 ^a^	10.21 ± 5.31 ^a^	11.08 ± 8.08 ^a^	0.75 ± 0.44 ^a^	31.31 ± 13.74 ^a^
	B	0.32 ± 0.57 ^a^	2.15 ± 0.99 ^bc^	5.06 ± 6.27 ^b^	4.25 ± 2.24 ^ab^	3.31 ± 3.04 ^ab^	19.31 ± 20.84 ^a^	16.82 ± 3.82 ^ab^	32.45 ± 20.54 ^b^	83.66 ± 38.47 ^b^
	C	0.27 ± 0.23 ^a^	1.27 ± 1.23 ^ab^	0.99 ± 0.41 ^a^	1.48 ± 0.31 ^a^	3.53 ± 1.15 ^ab^	6.71 ± 6.87 ^a^	19.49 ± 6.15 ^bc^	3.16 ± 1.66 ^a^	36.90 ± 11.68 ^a^
	D	0.07 ± 0.05 ^a^	2.61 ± 0.64 ^c^	2.74 ± 1.29 ^ab^	7.23 ± 7.40 ^b^	6.06 ± 3.62 ^b^	10.77 ± 7.34 ^a^	24.10 ± 1.62 ^c^	2.20 ± 0.38 ^a^	55.78 ± 14.66 ^a^
Statistical significance		*p* ˂ 0.05								
4th	A	1.67 ± 1.27 ^b^	3.03 ± 1.45 ^b^	4.29 ± 3.81 ^ab^	6.56 ± 4.87 ^a^	18.55 ± 16.46 ^b^	23.31 ± 10.57 ^b^	22.58 ± 5.69 ^bc^	1.06 ± 1.92 ^a^	81.05 ± 30.95 ^bc^
	B	0.97 ± 1.55 ^ab^	0.98 ± 1.12 ^a^	1.39 ± 0.89 ^a^	5.85 ± 4.71 ^a^	3.73 ± 2.57 ^a^	7.87 ± 4.46 ^a^	23.67 ± 10.86 ^c^	4.65 ± 4.58 ^b^	49.12 ± 23.46 ^ab^
	C	0.09 ± 0.05 ^a^	0.58 ± 0.38 ^a^	3.79 ± 0.62 ^ab^	4.28 ± 3.19 ^a^	3.45 ± 0.67 ^a^	11.77 ± 4.58 ^a^	7.32 ± 3.07 ^a^	1.30 ± 0.22 ^a^	32.59 ± 5.33 ^a^
	D	0.34 ± 0.44 ^a^	2.38 ± 1.26 ^b^	8.10 ± 7.37 ^b^	28.11 ± 28.35 ^b^	13.59 ± 12.59 ^ab^	28.36 ± 15.46 ^b^	15.55 ± 3.85 ^b^	3.39 ± 0.99 ^ab^	99.82 ± 61.65 ^c^
Statistical significance		*p* ˂ 0.05								
8th	A	2.31 ± 1.68 ^b^	1.35 ± 0.46 ^b^	2.87 ± 1.29 ^a^	4.60 ± 2.95 ^a^	6.11 ± 3.95 ^a^	4.95 ± 1.62 ^a^	7.84 ± 2.35 ^a^	21.34 ± 3.31 ^b^	51.37 ± 9.29 ^a^
	B	0.40 ± 0.06 ^a^	0.31 ± 0.09 ^a^	5.23 ± 4.10 ^a^	5.05 ± 3.58 ^a^	3.84 ± 0.38 ^a^	6.66 ± 3.60 ^a^	15.39 ± 11.04 ^a^	4.29 ± 5.44 ^a^	41.16 ± 20.47 ^a^
	C	2.77 ± 2.87 ^b^	0.74 ± 0.46 ^ab^	22.93 ± 15.04 ^b^	75.91 ± 52.06 ^b^	272.05 ± 217.83 ^b^	61.69 ± 20.53 ^b^	12.08 ± 6.69 ^a^	1.50 ± 0.57 ^a^	449.66 ± 297.81 ^b^
	D	0.14 ± 0.16 ^a^	1.41 ± 1.61 ^b^	17.66 ± 12.51 ^b^	45.96 ± 32.37 ^b^	3.11 ± 0.94 ^a^	46.12 ± 19.72 ^b^	16.56 ± 11.60 ^a^	1.79 ± 0.79 ^a^	132.74±56.44 ^a^
Statistical significance		*p* ˂ 0.05								

Statistically significant differences (*p* ˂ 0.05) between values for each day of storage (lower cases “^a^”, “^b^”, “^c^” in indexes in columns).

**Table 3:** Statistically significant differences (*p* ˂ 0.05) for a particular biogenic amine (lower case “a”, “b” in columns) depending on the sampling day and between biogenic amines (capital letters “*A*” to “*D*” in italic in rows) for the sampling day are given.

Day	Sample	Tryptamine		2-Phenylethylamine		Putrescine		Cadaverine		Histamine		Tyramine		Spermidine		Spermine		Sum of Bas
1st	A	a	*A*	a	*AB*	a	*BCD*	a	*BCD*	a	*BC*	a	*D*	a	*CD*	a	*AB*	a
4th		b	*A*	b	*AB*	a	*AB*	b	*ABC*	b	*BC*	b	*C*	b	*C*	a	*A*	b
8th		b	*AB*	a	*A*	a	*AB*	ab	*ABC*	a	*BCD*	a	*BC*	a	*CD*	b	*D*	a
1st	B	a	*A*	b	*AB*	a	*ABC*	a	*BCD*	a	*ABC*	b	*CD*	a	*D*	b	*D*	b
4th		a	*A*	a	*A*	a	*AB*	a	*BCD*	a	*ABC*	ab	*CD*	a	*D*	a	*ABC*	a
8th		a	*AB*	a	*A*	a	*CD*	a	*CD*	a	*CD*	a	*CD*	a	*D*	a	*BC*	a
1st	C	a	*A*	a	*AB*	a	*ABC*	a	*ABC*	a	*CD*	a	*BCD*	b	*D*	b	*BCD*	a
4th		a	*A*	a	*A*	a	*BC*	a	*BC*	a	*BC*	a	*C*	a	*C*	a	*AB*	a
8th		b	*A*	a	*A*	b	*BCD*	b	*CD*	b	*D*	b	*CD*	a	*ABC*	a	*AB*	b
1st	D	a	*A*	a	*B*	a	*AB*	a	*B*	ab	*BC*	a	*BC*	b	*C*	a	*AB*	a
4th		a	*A*	a	*AB*	a	*BC*	ab	*CD*	b	*BCD*	b	*D*	a	*CD*	b	*ABC*	ab
8th		a	*A*	a	*A*	b	*CD*	b	*D*	a	*ABC*	c	*D*	a	*BCD*	a	*AB*	b

**Table 4:** Qualitative and quantitative BA spectrum (in mg·kg^−1^) for three batches and for one batch for groups C and D/8th day of sampling in a detailed view.

Day/Sample		Tryptamine	2-Phenyl-Ethylamine	Putrescine	Cadaverine	Histamine	Tyramine	Spermidine	Spermine	Sum of Bas
8th	3/C	3.67 ± 2.77 ^a^*^A^*	0.83 ± 0.51 ^a^*^A^*	27.96 ± 14.04 ^b^*^A^*	97.22 ± 41.03 ^b^*^A^*	354.74 ± 185.67 ^b^*^B^*	68.68 ± 18.96 ^b^*^A^*	8.64 ± 2.91 ^a^*^A^*	1.58 ± 0.64 ^a^*^A^*	563.32 ± 252.61 ^b^
	1/C	0.05 ± 0.00 ^a^*^A^*	0.46 ± 0.01 ^a^*^AB^*	7.83 ± 0.15 ^a^*^C^*	11.97 ± 0.03 ^a^*^D^*	23.98 ± 0.93 ^a^*^F^*	40.71 ± 0.34 ^a^*^G^*	22.39 ± 0.10 ^b^*^E^*	1.28 ± 0.15 ^a^*^B^*	108.67 ± 1.67 ^a^
	S. s.	*	*	*p* ˂ 0.05	*p* ˂ 0.05	*p* ˂ 0.05	*p* ˂ 0.05	*p* ˂ 0.05	*	*p* ˂ 0.05
	3/D	0.16 ± 0.18 ^a^*^A^*	1.67 ± 1.80 ^a^*^AB^*	20.30 ± 13.56 ^a^*^B^*	58.93 ± 26.15 ^b^*^C^*	3.36 ± 0.97 ^a^*^AB^*	52.99 ± 17.96 ^a^*^C^*	12.89 ± 11.15 ^a^*^AB^*	1.73 ± 0.91 ^a^*^AB^*	152.04 ± 52.01 ^b^
	1/D	0.06 ± 0.01 ^a^*^A^*	0.62 ± 0.02 ^a^*^A^*	9.75 ± 0.35 ^a^*^D^*	7.04 ± 0.19 ^a^*^C^*	2.36 ± 0.10 ^a^*^B^*	25.51 ± 0.59 ^a^*^E^*	27.59 ± 0.25 ^a^*^F^*	1.94 ± 0.05 ^a^*^B^*	74.87 ± 1.12 ^a^
	S. s.	*	*	*	*p* ˂ 0.05	*	*p* ˂ 0.05	*	*	*p* ˂ 0.05

Statistically significant differences (*p* ˂ 0.05) for a particular biogenic amine (lower case “^a^”, “^b^” in columns) and between biogenic amines (capital letters “*^A^”* to *“^G^”* in italic in rows) depending of number of batches are given. * values are without statistically significant differences (S. s.).

**Table 5:** Sensory attributes evaluation for nigiri sushi meal with not smoked and smoked samples of tuna and salmon fillets.

Sushi	Treatment	Salt %	Saltiness	Bitterness	Juiciness	Consistency
tuna	not smoked	0		16.25 ± 23.13	32.8 ± 36.15	34.15 ± 36.88
		2	55.55 ± 20.45	29.6 ± 38.57	16.7 ± 21.72	9.6 ± 8.45
		3		20.00 ± 30.60	27.45 ± 25.43	9.1 ± 10.93
	smoked	0		20.09 ± 30.71	26.18 ± 29.02	21.95 ± 26.67
		2	59.68 ± 17.84	23.45 ± 30.08	18.36 ± 20.27	9.64 ± 6.47
		3		17.91 ± 24.48	25.00 ± 23.34	14.45 ± 15.05
salmon	not smoked	0		14.6 ± 10.87	18.25 ± 6.88	18.1 ± 26.23
		2	53.9 ± 23.68	11.25 ± 9.58	13.55 ± 7.12	9.2 ± 5.71
		3		18.1 ± 14.59	20.25 ± 7.38	11.4 ± 5.21
	smoked	0		20.80 ± 17.32	21.25 ± 18.92	19.10 ± 18.58
		2	48.22 ± 20.33	18.35 ± 19.50	12.10 ± 6.48	7.85 ± 4.54
		3		17.60 ± 12.32	22.00 ± 13.19	16.60 ± 13.51

Saltiness (2% salt addition) was evaluated by respondents’ comparison with sushi samples prepared without salt (0 point) and with 3% salt content (100 points) added to rice.

**Table 6:** The content of polycyclic aromatic hydrocarbons (PAH) in μg·kg^−1.^

Fish	Treatment	B(a)a	Chr	B(b)f	B(a)p	Sum of Pah
tuna	not smoked	<0.24	<0.28	<0.28	<0.27	*
	smoked	1.84	1.10	0.52	0.76	4.22
salmon	not smoked	<0.24	<0.28	<0.28	<0.27	*
	smoked	<0.24	0.77	0.37	0.60	1.74

B(a)A—benzo[a]anthracene; CHr—chrysene; B(b)F—benzo[b]fluoranthene; B(a)P—benzo[a]pyrene; * values under limit of detection (LOD).

**Table 7:** Chemical composition of sashimi tuna fillets and salmon fillets with skin (in %).

Samples	Protein	Fat	Dry Matter
tuna	29.02 ± 0.06	0.83 ± 0.01	30.09 ± 0.70
salmon	20.46 ± 0.98	23.46 ± 0.96	43.63 ± 0.00

## Appendix A

### Appendix A.1. Legislation and Procedures

1. Commission Regulation (EC) No 2073/2005 of 15 November 2005 on microbiological criteria for foodstuffs. Available online. https://eur-lex.europa.eu/legal-content/EN/TXT/?qid=1565694175620&uri=CELEX:02005R2073-20190228 (accessed on 28 February 2019).
2. Regulation (EC) No 853/2004 of the European Parliament and of the Council of 29 April 2004 laying down specific hygiene rules for food of animal origin. Available online: https://eur-lex.europa.eu/legal-content/EN/TXT/?qid=1565693935604&uri=CELEX:02004R0853-20190101 (accessed on 1 January 2019).
3. International Organization for Standardization ISO 8589:2008 Sensory analysis—General guidance for the design of test rooms
4. International Organization for Standardization ISO 937:1978 Meat and meat products—Determination of nitrogen content (Reference method)
5. International Organization for Standardization ISO 1443:1973 Meat and meat products—Determination of total fat content. (Reference method)
6. International Organization for Standardization ISO 1442:1997 Meat and meat products—Determination of moisture content (Reference method)
7. Regulation (EC) No 1333/2008 of the European Parliament and of the Council of 16 December 2008 on food additives. Available online: https://eur-lex.europa.eu/legal-content/EN/TXT/?qid=1565693786967&uri=CELEX:02008R1333-20190618 (accessed on 18 July 2019).
8. Commission Regulation (EC) No 1881/2006 of 19 December 2006 setting maximum levels for certain contaminants in foodstuffs. https://eur-lex.europa.eu/legal-content/EN/TXT/?qid=1565694263939&uri=CELEX:02006R1881-20180319 (accessed on 19 March 2018).
